# A Pilot Study on the Efficacy of Artificial Intelligence-Driven Monocular Three-Dimensional Conversion for Endoscopic Spatial Perception

**DOI:** 10.7759/cureus.111858

**Published:** 2026-06-30

**Authors:** Yosuke Sato, Kosuke Tanaka

**Affiliations:** 1 Neurosurgery, Showa University, Tokyo, JPN; 2 Brain Function Analysis and Digital Medicine Research Institute, Showa University, Tokyo, JPN

**Keywords:** artificial intelligence, depth perception, endoscopic surgery, medical simulation, spatial perception, three-dimensional conversion

## Abstract

Background: Monocular two-dimensional endoscopes inherently lack depth perception, significantly increasing the cognitive load during surgical procedures. Artificial intelligence has the potential to convert two-dimensional video feeds into stereoscopic three-dimensional views in real time, yet its depth-dependent limitations remain underexplored. This pilot study evaluated the efficacy and spatial limitations of an artificial intelligence-driven monocular three-dimensional conversion system.

Methods: Six participants performed a surgical target-grasping task within a dry box simulator under three distinct visual conditions: a monocular two-dimensional baseline, an artificial intelligence-generated three-dimensional system, and a native stereoscopic three-dimensional system. Targets were placed at three specific working distances of 3 cm, 5 cm, and 10 cm, yielding a total of 90 independent trials.

Results: The overall grasping success rate was 33.3% for the two-dimensional baseline, 52.2% for the artificial intelligence system, and 68.9% for the native three-dimensional system. Statistical analysis confirmed that the artificial intelligence conversion significantly improved the success rate over the standard two-dimensional environment. Furthermore, the artificial intelligence system drastically reduced the average task completion time to 5.25 seconds compared to 7.93 seconds in the two-dimensional setting. A distance-based sub-analysis revealed a notable physical limitation. The artificial intelligence system provided exceptional spatial support, comparable to native three-dimensional systems, at close working distances of 3 cm and 5 cm. However, at a deeper distance of 10 cm, the monocular depth cues diminished, leading to a decline in estimation accuracy.

Conclusions: Real-time artificial intelligence-driven three-dimensional conversion successfully enhances spatial perception and operational efficiency in surgical environments. While it serves as a highly effective visual assist tool at standard close working distances, operators must remain aware of its limitations in deeper operative spaces.

## Introduction

Endoscopic and laparoscopic surgeries have revolutionized minimally invasive medicine, significantly reducing patient trauma and accelerating postoperative recovery [1,2]. However, the inherent reliance on monocular two-dimensional (2D) displays fundamentally deprives the operator of binocular depth cues, leading to a critical loss of natural spatial perception [3]. This lack of depth information significantly increases the cognitive load, prolongs the learning curve for novice surgeons, and poses potential safety risks during delicate spatial maneuvers such as suturing, precise grasping, or dissection in confined anatomical spaces [4].

To address these limitations, dedicated three-dimensional (3D) endoscopic systems equipped with dual-lens hardware have been developed and increasingly introduced into clinical practice [1]. Multiple studies have demonstrated that native hardware-based 3D visualization significantly improves depth perception, reduces operative time, and enhances overall surgical precision compared to standard 2D systems [1]. Despite these proven advantages, the widespread adoption of native 3D endoscopes is hindered by substantial physical and economic constraints. The necessity of housing two distinct optical sensors inevitably increases the outer diameter of the endoscope, limiting its maneuverability in narrow anatomical corridors and potentially increasing patient discomfort [5]. Furthermore, these proprietary dual-lens systems are highly expensive and can induce visual fatigue or cybersickness in operators due to vergence-accommodation conflicts during prolonged procedures [1].

Recent advancements in artificial intelligence (AI) and computer vision, particularly in the domain of monocular depth estimation (MDE), offer a promising software-based alternative to overcome the physical limitations of dual-lens hardware. State-of-the-art deep learning architectures, including self-supervised networks and generative adversarial networks, have demonstrated the theoretical capability to reconstruct highly accurate 3D topographies from a single 2D camera feed in real time [6]. In the context of medical imaging, AI-driven depth estimation has been increasingly explored to extract temporal and spatial consistency from standard endoscopic video streams without requiring any hardware modifications [7]. These software-driven stereoscopic conversions have the potential to provide the spatial benefits of native 3D systems while utilizing existing, ultra-thin monocular endoscopes [8,9]. This approach could theoretically democratize the use of 3D visualization across various endoscopic disciplines [10].

Despite the rapid evolution of these algorithms, there remains a critical gap in evaluating their practical efficacy and physical limitations in surgical simulation environments. Specifically, the accuracy of AI-generated depth perception at varying working distances remains underexplored in the literature. Therefore, this pilot study aims to evaluate the efficacy of a novel AI-driven monocular 3D conversion system. By utilizing a dry box simulator and a standardized target-grasping task at specific working distances (3 cm, 5 cm, and 10 cm), we investigate whether real-time AI stereoscopic conversion can improve operational efficiency and spatial perception compared to a conventional 2D baseline, while simultaneously identifying the distance-dependent limitations inherent to monocular depth inference.

## Materials and methods

Experimental design and simulation environment

**Figure 1:**
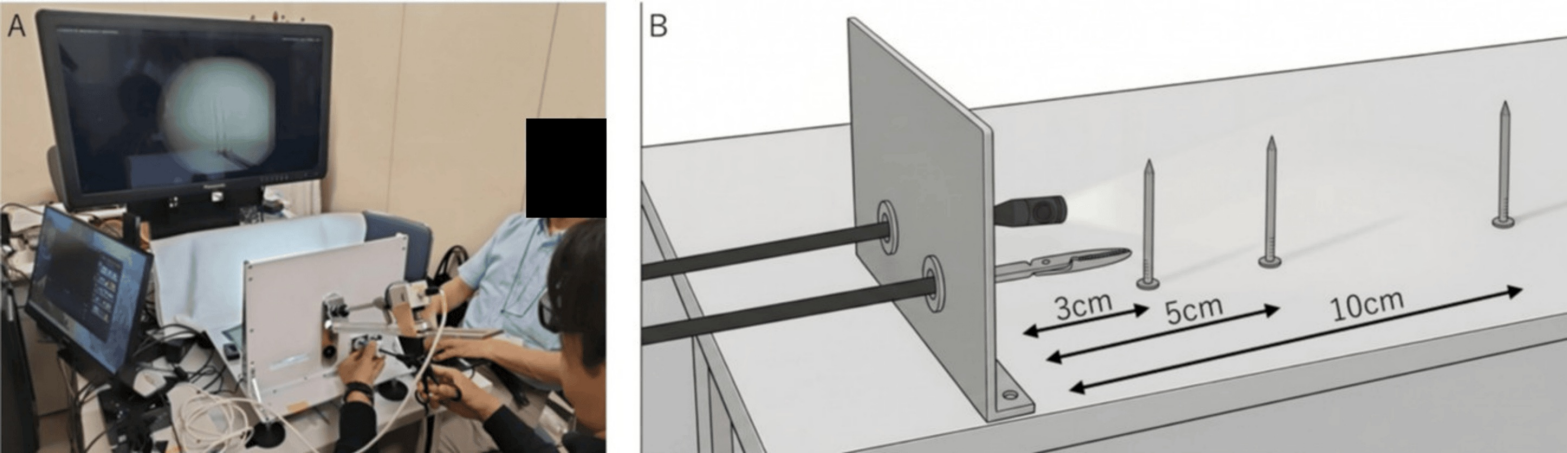
Experimental setup of the dry box simulator and target-grasping task. (A) Photograph of a participant wearing 3D glasses and operating the grasping forceps in front of a 3D medical monitor. (B) Schematic diagram of the open experimental area beyond the single access port wall. Three 70 mm inverted nails serve as targets, positioned at standardized working distances of 3 cm, 5 cm, and 10 cm from the endoscope tip. 3D: three-dimensional. The schematic diagram (B) was created manually using Microsoft PowerPoint (Microsoft Corporation, Redmond, Washington, USA).

Six non-medical volunteers participated in a standardized target-grasping task in a controlled dry-box simulator environment. The task was designed to strictly evaluate spatial perception and depth estimation without the bias of prior endoscopic training, utilizing an established methodology for assessing endoscopic visualization systems [5]. Participants were tasked with grasping a target placed at three specific working distances from the endoscope tip: 3 cm, 5 cm, and 10 cm. The experimental environment, including the physical arrangement of the dry box, endoscope positioning, and target alignment, was carefully standardized across all trials to ensure reproducibility, as illustrated in Figure 1.

Visual conditions and artificial intelligence (AI)-driven conversion system

**Figure 2:**
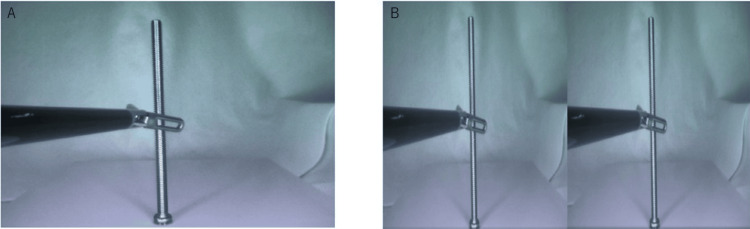
Real-time artificial intelligence-driven monocular three-dimensional conversion process. Visual demonstration of the software-based transformation. (A) The original monocular two-dimensional (2D) input from the endoscope. (B) The corresponding stereoscopic three-dimensional (3D) output generated in a side-by-side format by the proprietary artificial intelligence algorithm. Image Credit: Yosuke Sato.

Each participant performed the grasping task under three distinct visual conditions: a conventional monocular two-dimensional (2D) baseline, a novel artificial intelligence (AI)-generated three-dimensional (3D) system, and a native hardware-based stereoscopic 3D system. To eliminate learning effects and task familiarization bias, the sequence in which these three visual conditions were presented was fully randomized and counterbalanced for each participant. The AI-driven system utilized a proprietary real-time algorithm to process the standard monocular 2D video feed and generate a stereoscopic view presented in a side-by-side format (Figure 2). Due to pending patent considerations regarding the core technology, the specific neural network architecture and depth-inference algorithms are omitted from this report. However, the computational pipeline was deployed on an NVIDIA DGX SPARK hardware environment, utilizing TensorRT optimizations, CUDA Graphs, and Zero-Copy memory architectures to maximize inference speed. The system processed Full HD (1920x1080) input streams down to a 1024x1024 tensor space for real-time stereoscopic rendering, maintaining strict synchronization with the native camera frame rate to minimize latency. The grasping task was repeated up to a maximum of five attempts per distance under each visual condition, yielding a total of 90 independent trials.

Statistical analysis

The primary endpoints evaluated were the overall grasping success rate and the mean task completion time. A repeated-measures analysis of variance (RM-ANOVA) was utilized to evaluate differences in mean completion times across the visual conditions. Overall grasping success rates were analyzed using an omnibus Chi-square test, followed by post-hoc pairwise comparisons using the Bonferroni correction. To assess the magnitude of the observed differences, effect sizes were calculated using Cramer's V for categorical data and partial eta-squared (ηp2) for the RM-ANOVA. Furthermore, 95% confidence intervals (CIs) were determined for overall success rates. The level of statistical significance was set at p < 0.05. All statistical analyses were performed using Microsoft Excel (Microsoft Corporation, Redmond, WA, USA).

## Results

Task performance and statistical outcomes

The primary endpoints evaluated were the overall grasping success rate and the mean task completion time. Across all distances, the overall success rate was 33.3% (30/90) for the 2D baseline, 52.2% (47/90) for the AI-driven 3D system, and 68.9% (62/90) for the native 3D system. Furthermore, the AI system drastically reduced the average task completion time to 5.25 seconds, representing a substantial improvement over the 7.93 seconds recorded in the standard 2D setting. A repeated-measures analysis of variance (RM-ANOVA) confirmed that the AI-driven conversion significantly improved operational efficiency compared to the 2D baseline. A detailed breakdown of the grasping success rates, mean completion times, and corresponding statistical p-values across all three working distances and visual conditions is summarized in Table 1.

**Table 1:** Summary of target-grasping success rates and mean completion times across three visual conditions and working distances. *Denotes statistical significance in post-hoc pairwise comparisons (Bonferroni correction) for overall success rates against the Monocular 2D baseline. An omnibus test revealed a significant difference in overall success rates across all three visual conditions (X²(2) = 22.79, p < 0.001, Cramer's V = 0.29). The 95% confidence intervals (CIs) for overall success rates were (23.6%, 43.1%) for 2D, (41.9%, 62.5%) for AI-driven 3D, and (59.3%, 78.5%) for Native 3D. Two-way repeated-measures ANOVA demonstrated a significant main effect of the visual condition on mean completion time (F(2, 10) = 5.82, p = 0.021, ηp2 = 0.54). The level of statistical significance for all tests was established at p < 0.05.

Visual condition	Working distance	First-attempt success rate (%)	Overall success rate (%)	p-value (vs. 2D)	Mean completion time (s)
Monocular 2D (Baseline)	3 cm	0.0% (0/6)	40.0% (12/30)	-	8.43
	5 cm	33.3% (2/6)	40.0% (12/30)	-	7.3
	10 cm	16.7% (1/6)	20.0% (6/30)	-	8.07
	Overall	16.7% (3/18)	33.3% (30/90)	Reference	7.93
AI-driven 3D (Proposed)	3 cm	66.7% (4/6)	63.3% (19/30)	-	4.53
	5 cm	50.0% (3/6)	60.0% (18/30)	-	5.03
	10 cm	33.3% (2/6)	33.3% (10/30)	-	6.2
	Overall	50.0% (9/18)	52.2% (47/90)	0.011*	5.25
Native 3D (Hardware)	3 cm	33.3% (2/6)	60.0% (18/30)	-	5.1
	5 cm	83.3% (5/6)	73.3% (22/30)	-	4.33
	10 cm	83.3% (5/6)	73.3% (22/30)	-	5.47
	Overall	66.7% (12/18)	68.9% (62/90)	<0.001*	4.97

Distance-dependent spatial limitations

**Figure 3:**
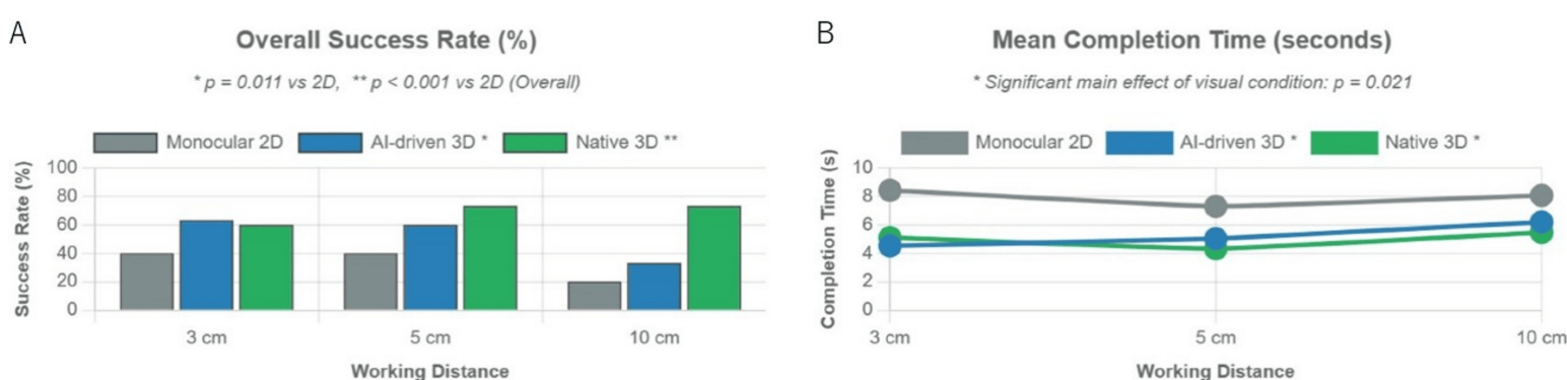
Comparison of target-grasping success rates and mean completion times across varying working distances. (A) Overall success rate: Bar chart illustrating the total grasping success rates (%) across three standardized working distances (3 cm, 5 cm, and 10 cm). Statistical significance for overall success rates compared to the monocular 2D baseline is denoted by *p = 0.011 and **p < 0.001. (B) Mean completion time: Line graph representing the average task completion times (seconds) across the same working distances. For mean completion times, repeated-measures ANOVA demonstrated a significant main effect of the visual condition (*p = 0.021). 2D: Two-dimensional; 3D: three-dimensional; AI: artificial intelligence; RM-ANOVA: repeated-measures analysis of variance.

A distance-based sub-analysis revealed distinct operational characteristics inherent to the AI-driven monocular conversion. At close working distances of 3 cm and 5 cm, the AI system provided exceptional spatial support, resulting in success rates and task completion times that were highly comparable to those achieved with the native stereoscopic 3D system. However, at the deeper working distance of 10 cm, the AI-generated monocular depth cues diminished, leading to a noticeable decline in depth estimation accuracy and a subsequent increase in task completion time. The direct correlation between working distance, grasping success rates (Figure 3A), and completion times (Figure 3B) across the three visual conditions is graphically depicted.

## Discussion

The fundamental challenge in conventional monocular endoscopic surgery remains the inherent lack of binocular depth perception, which forces operators to rely on secondary visual cues such as shadows, texture gradients, and instrument occlusion [3]. Our findings demonstrate that real-time artificial intelligence (AI)-driven three-dimensional (3D) conversion significantly enhances spatial awareness, bridging the performance gap between traditional two-dimensional (2D) displays and dedicated stereoscopic hardware. The AI system improved the overall grasping success rate to 52.2%, compared to the 33.3% baseline of the 2D system, while simultaneously reducing the mean task completion time from 7.93 seconds to 5.25 seconds. These improvements in operational efficiency and precision are critical in clinical settings, where prolonged operative times and elevated cognitive loads directly correlate with surgical fatigue and procedural errors [1,3].

While native 3D endoscopes provide superior stereopsis, their widespread adoption is frequently constrained by the physical necessity of dual-lens configurations, which inherently increase the scope diameter and limit maneuverability in constrained anatomical spaces [1,5]. By utilizing a purely computational approach to extract depth information from a standard monocular feed, our system circumvents these physical hardware constraints. Recent advancements in monocular depth estimation have proven that deep learning architectures can accurately infer spatial geometries [6,7]. Our results validate this theoretical potential in a practical simulation, showing that the AI conversion can provide exceptional spatial support that rivals native 3D systems at close working distances of 3 cm and 5 cm. This suggests that software-based stereoscopic conversion could offer a highly scalable and cost-effective alternative to bulky hardware solutions without sacrificing instrument maneuverability [8,9].

Despite the promising performance at close range, our distance-based sub-analysis revealed a notable physical limitation inherent to monocular depth inference. At a deeper working distance of 10 cm, the AI system exhibited a decline in depth estimation accuracy, resulting in lower grasping success rates compared to the native 3D system. This degradation is likely attributable to the diminishing quality of monocular depth cues, such as illumination gradients and textural resolution, which AI models heavily rely upon to extrapolate depth in the absence of true binocular parallax [9,10]. In deeper operative fields, the lack of sufficient geometric and photometric variance challenges the algorithm's ability to maintain spatial and temporal consistency [7]. Acknowledging this boundary is vital for patient safety, as operators must be aware that software-driven depth perception is highly reliable at standard close ranges but requires caution in deeper cavities. Future iterations of the algorithm will necessitate training on more diverse, distance-calibrated datasets and the integration of temporal consistency refinements to extend the effective range of monocular stereopsis [6,7].

This study has several limitations that must be acknowledged. First, the sample size was relatively small (n = 6), and the participants were non-medical volunteers. While this design intentionally eliminated prior endoscopic bias to purely evaluate visual perception, future studies involving experienced surgeons are necessary to validate these findings in clinical workflows. The operator's baseline experience likely influences the magnitude of the system's benefit; novices who struggle with the loss of binocular cues may experience pronounced improvements, whereas experienced surgeons who have adapted to secondary monocular cues may find the AI assistance less impactful, though potentially beneficial for reducing cognitive fatigue. Therefore, generalizing these pilot results to real clinical practice requires cautious interpretation. Second, the experiments were conducted in a controlled dry box simulator, which lacks the dynamic complexities of in vivo environments, such as bleeding, smoke generation, and respiratory tissue movement. These real-world factors could potentially impact the algorithm's real-time depth inference and temporal consistency. Finally, the target-grasping task utilized static objects; evaluating dynamic tracking of moving targets will be essential for a comprehensive assessment of the system's spatial capabilities.

## Conclusions

Real-time artificial intelligence-driven monocular three-dimensional conversion offers a highly promising software-based solution to overcome the depth perception limitations inherent in conventional two-dimensional endoscopy. Our findings demonstrate that this computational approach significantly improves spatial awareness and operational efficiency, achieving performance comparable to native hardware-based stereoscopic systems at close working distances. While the system effectively circumvents the physical constraints of dual-lens endoscopes, operators must remain cognizant of its distance-dependent limitations, particularly the degradation of depth estimation accuracy at deeper operative fields. Future advancements targeting temporal consistency and distance-calibrated training data will be crucial to expanding the robust operative range of monocular stereopsis, ultimately democratizing safe and efficient three-dimensional visualization across diverse minimally invasive surgical disciplines.
